# Anatomic 3D-Printed Endoprosthetic With Multiligament Reconstruction After En Bloc Resection in Giant Cell Tumor of Distal Radius

**DOI:** 10.5435/JAAOSGlobal-D-20-00178

**Published:** 2021-02-22

**Authors:** Vanasiri Kuptniratsaikul, Pobe Luangjarmekorn, Chris Charoenlap, Chindanai Hongsaprabhas, Pravit Kitidumrongsook

**Affiliations:** From the Department of Orthopaedics, Faculty of Medicine, Chulalongkorn University, Bangkok, Thailand.

## Abstract

A 34-year-old woman was diagnosed with a giant cell tumor of the right distal radius with extensive articular invasion. After en-bloc resection of 5.5 cm of the distal radius, reconstruction was done with three-dimensional printing custom-made distal radius prosthesis. In addition, a multiligament reconstruction was done to prevent postoperative radiocarpal subluxation and imitate the native distal radius. At 18 months, the range of motion was 20° dorsiflexion, 10° palmar flexion, 10° supination, and 60° pronation. Her grip strength was 60% compared with the contralateral side. No complications were seen during this 2-year follow-up. We present the combined 3-dimensional printed custom-made prosthetic with multiligament reconstruction as an innovative method without postoperative complication at 2 years.

Giant cell tumor (GCT) is considered benign in metastasis but behaves aggressive locally. This tumor localizes around 2% in the hand,^1,2^ and the distal radius is the third most common location.^3^ En bloc excision to completely remove the tumor is indicated in Campanacci grade II/III GCT because an extremely high rate of local recurrence exits after curettage only.^4,5^ Skeletal reconstruction of the extended bone defect is remarkably challenging to achieve excellent outcome on both functional demand and cosmesis with the lowest complication. Various techniques have been proposed such as ulnar translocation^6^ and wrist arthrodesis^7–9^ that are suitable for cases where the tumor extends into the carpal bones or in revision cases. However, these procedures result in a loss of motion. Nonvascularized^10^ or vascularized^11–13^ autograft and allograft^14–17^ are biologic replacements, yet wrist instability and degenerative changes remain problematic. The main advantages for reconstruction with a prosthetic replacement^18–20^ are anatomical restoration of a large defect, earlier recovery, and avoidance of donor site morbidity. This article presents a reconstruction technique using a three-dimensional (3D)-printed custom-made endoprosthesis with multiple ligament reconstruction. We report postoperative functional results and complications of the first patient who underwent this procedure.

## Case Report

**Figure 1:**
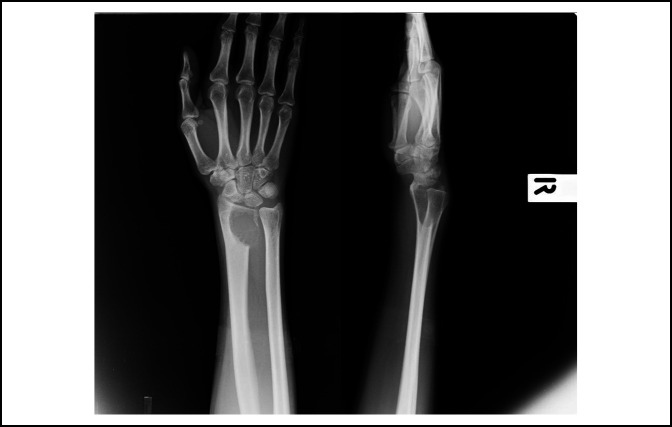
Radiographs demonstrating an osteolytic lesion with ill-defined margin of the distal radius on PA and lateral views.

**Figure 2:**
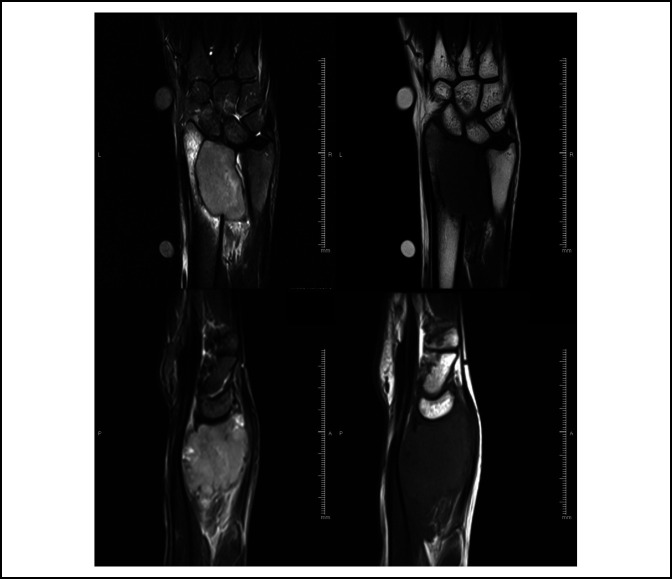
Magnetic resonance imaging demonstrating coronal and sagittal views in T1 and T2 of the distal radius.

A 34-year-old right-handed woman presented with a 6-month history of a painful mass (3 × 5 cm) on her right wrist. At initial presentation, the patient had 0° of wrist flexion, 0° extension, 0° supination, and had only 10° of pronation remaining. Radiographs demonstrated an osteolytic lesion in the distal radius with ill-defined margins (Figure 1). After the pathological confirmation of grade III GCT, reconstructive options were discussed. The patient agreed to reconstruction with a custom-made endoprosthesis and multiligament reconstruction technique because of the shorter recovery time and preserved postoperative motion. Preoperative investigations included radiographs of bilateral wrist and chest x-rays; bilateral wrists 3D CT scan and right wrist magnetic resonance imaging (MRI) were done when appropriate (Figure 2). No lung metastasis was found.

## Prosthesis

**Figure 3:**
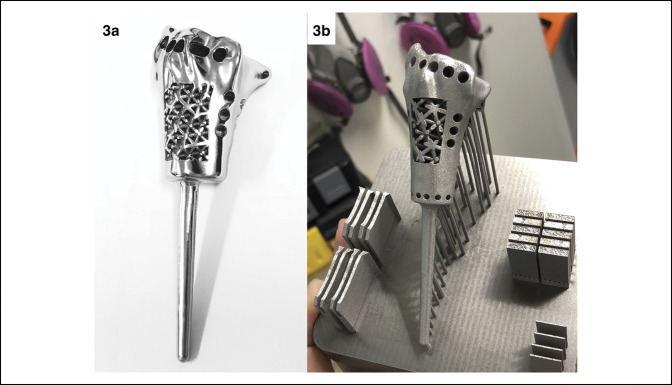
Figures demonstrating a custom-made distal radial endoprosthesis. **A**, Designed preoperatively by our developed technique via three-dimensional CT. **B**, Postprocessing model.

A custom-made endoprosthesis of the right distal radius with appropriate dimensions obtained from preoperative radiographs was used. Both wrists were scanned by a CT imaging system (GE Revolution 256; GE Healthcare) and MRI (Achieva 1.5T; Philips Healthcare) imaging equipment. The obtained sections (DICOM files) were uploaded into OsiriX MD Viewer Software (Pixmeo), and Solidworks (Dassault Systèmes) was used as a computer-aided design program for prototyping. Finally, a 3D-printed model was generated with Titanium (Ti-6AI-4V grade) from the Mlab 200R, a direct metal laser melting metal machine (Concept Laser) (Figure 3).

## Surgical Technique and Postoperative Management

The institutional review board (IRB) of the Faculty of Medicine, Chulalongkorn University approved this study (certificate No. 025/2020, IRB No. 488/63). The patient was placed under general anesthesia with the contralateral leg and arm prepped and draped appropriately. Her right arm was elevated before inflation of the tourniquet without exsanguination. A standard longitudinal dorsal wrist incision was made overlying the radius incooperating with and extending from previous biopsy site. Extensor digitorum communis tendons were retracted to the ulnar side, and extensor pollicis longs was retracted to the radial side. Dorsal wrist capsule was identified. During en bloc tumor resection, the distal edge of the dorsal wrist capsule was preserved. The dorsal radiolunotriquetral ligament was identified and dissected to its attachment on the dorsal lip of the distal radius to be reconstructed later. Although preserving the palmar radiolunate ligament and volar capsule, En bloc resection of the tumor was done to the preoperatively determined level based on the extent of the bone and soft-tissue involvement from CT and MRI. An additional transverse osteotomy at the shaft of radius was done to accommodate the custom-made prosthesis.

**Figure 4:**
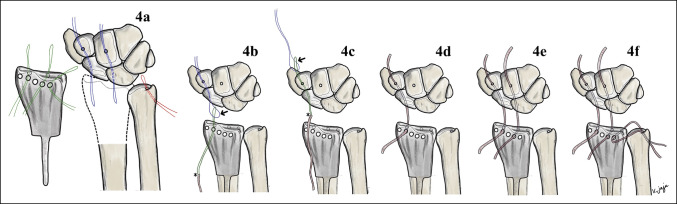
Diagrams demonstrating reconstruction of radiocarpal and distal radioulnar ligaments. **A**, Two blue wire loops were for radiocarpal reconstruction and single red wire loop was for radioulnar ligament reconstruction. **B**, Tendon grafts were tied with the green sutures at the dorsum of the prosthesis (asterisk) and blue wire loops were used for retrieving the green suture (arrow sign) from the volar side. **C**, Blue wire loops were retrieving green sutures (arrow sign). **D**, Tendon grafts were pulled from the dorsum of the prosthesis through the volar of the carpus and back to the dorsum of distal carpal row. **E**, Two tendon grafts were passed to reconstruct radiocarpal ligament. **F**, Another single tendon graft was passed to reconstruct distal radioulnar ligament.

For reconstruction of radiocarpal and distal radioulnar ligaments, two vertical tunnels at the distal carpal row (capitate and trapezoid bone) and one oblique tunnel at the base of ulnar styloid were made with a 2.5 mm drill-bit. Two wire loops (shown in blue in Figure 4A) were passed into the hole from dorsal to volar. The tips of wire loops were left for retrieving the tendon graft at the volar side of distal carpal row. Another wire loop (shown in red in Figure 4A) for the distal radioulnar ligament reconstruction was passed from proximal to distal, through the hole at the base of ulnar styloid. The tip of the wire loop was also left proud for retrieving the tendon. Before prosthesis insertion, three guide sutures (Ethibond 2-0) were placed into the holes of the distal radius prosthesis to help pass the tendon graft through the prosthesis (shown in green in Figure 4A).

A custom-made distal radial endoprosthesis with anatomical dimensions obtained from preoperative 3D CT was used for reconstruction. This prosthesis was inserted into the intramedullary canal of the proximal radial shaft with cement. The intramedullary canal of the radius was prepared by reaming and irrigating with normal saline. After canal drying, bone cement was inserted into the medullary canal by syringe injection and digital packing. Then, the prosthesis was inserted and held steady until the cement was set.

Next, three strips of autogenous tendon graft were prepared for reconstruction of the capsuloligamentous structures of the remaining carpal bones to the endoprosthesis. In this patient, we had to use a hamstring graft because of absence of palmaris longus and plantaris tendons. The hamstring tendon graft was longitudinally split to reduce the size (final diameter 2.0 mm) to facilitate pass through the 2.5 mm drill hole.

The strips of tendon grafts were tied with the guide sutures at the dorsum of the prosthesis (shown by asterisk sign in Figure 4B). Gentle traction to the wrist to assess to the wire loop and guide suture at the space between prosthesis and carpal bone was done. Then, the wire loops at the volar side of the carpus were used for retrieving the guide suture with tendon graft (shown by arrow sign in Figure 4B). By this method, the tendon grafts were pulled from the dorsum of the prosthesis through the volar of the carpus and back to the dorsum of distal carpal row without need to open the volar incision (Figure 4, C and D).

**Figure 5:**
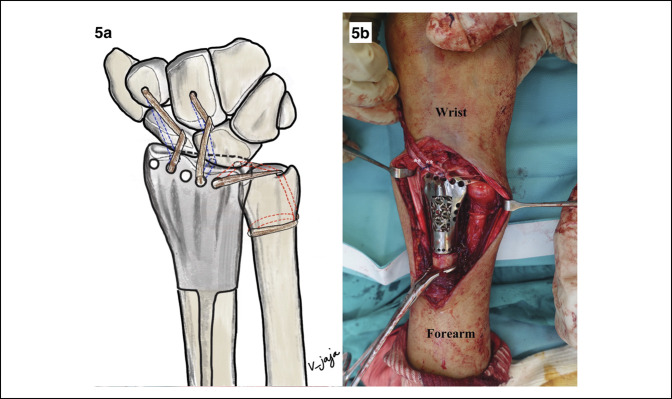
**A**, Diagram and (**B**) intraoperative procedure demonstrating multiligament reconstruction of radiocarpal joint (double white asterisks) and radioulnar joint (single black asterisk) by autogenous tendon grafts harvested from hamstring tendon.

The most-radial hole of the prosthesis was tensioned first, followed by central and distal radioulnar joint holes (Figure 4, E and F). Before tightening all ligaments, confirmation that the radiocarpal and distal radioulnar joints were reduced in normal position is needed. For radiocarpal joint, the stability was preferred over motion. Tension of the ligament at the radiocarpal joint was set at 30° wrist extension. We intentionally set the tension of the radiocarpal ligament as tight as we could to guarantee stable radiocarpal joint, although some stiffness might occur. For distal radioulnar joint, tension of distal radioulnar ligament reconstruction was set at the neutral pronosupination position of the wrist and confirmed that the ligaments were not overtensioned. As a result, intraoperative range of motion was 45° wrist flexion, 45° wrist extension after radiocarpal ligaments were tensioned, and full pronosupination was observed after distal radioulnar ligament was tightened (Figure 5).

**Figure 6:**
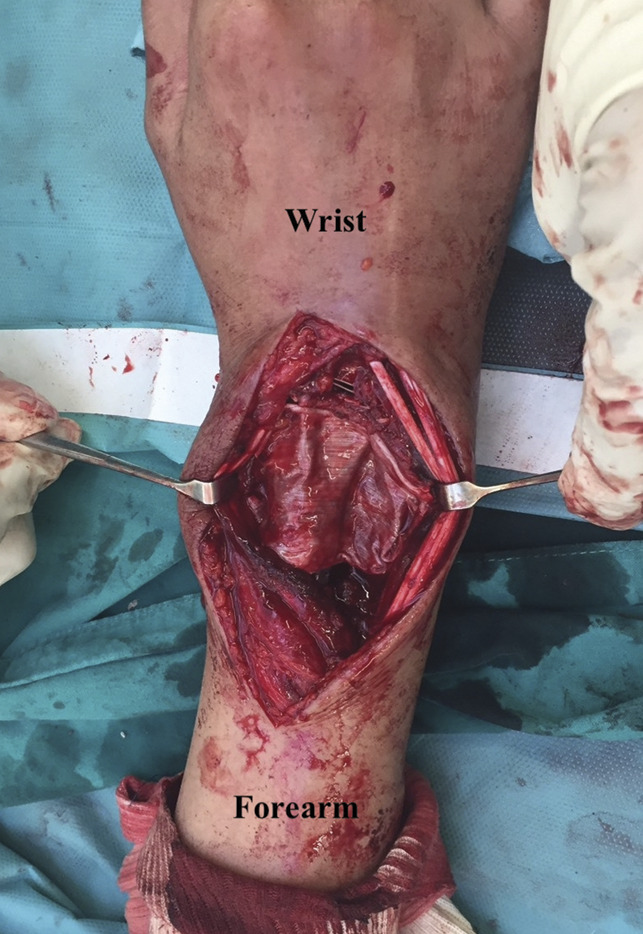
Figure demonstrating that extensor retinaculum was reconstructed over the endoprosthesis with tensor fascia lata.

Finally, the tensor fascia lata was harvested from the left thigh to reconstruct the dorsal wrist capsule over the endoprosthesis to prevent irritation or rupture of the extensor tendons (Figure 6). Final alignment was checked under a fluoroscope. The tourniquet was deflated, and meticulous hemostasis was ensured before closing. The wound was tightly closed over a suction drain placed in line with the incision.

Postoperatively, the limb was immobilized in an above-elbow cast for 4 weeks, then active range of motion wrist exercises were allowed, and intensity was gradually increased.

## Results

**Figure 7:**
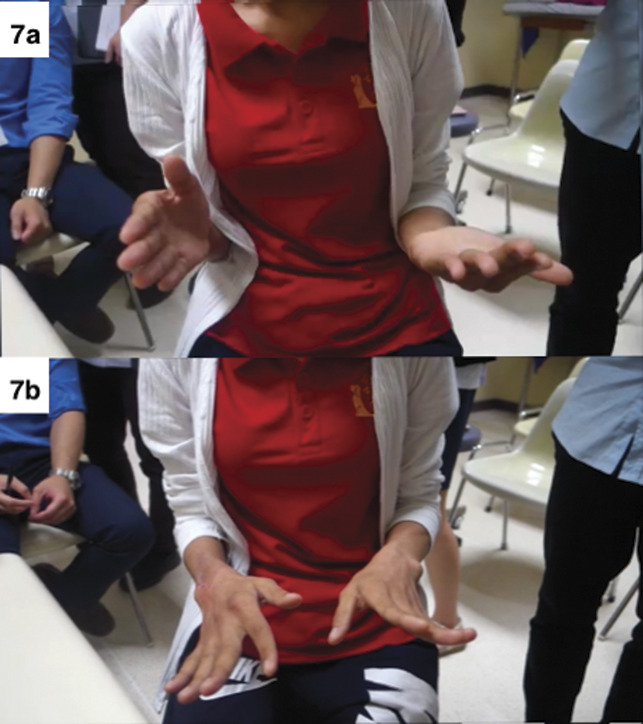
Functional photographs of the patient after 18 months. **A**, Supination. **B**, Pronation.

**Figure 8:**
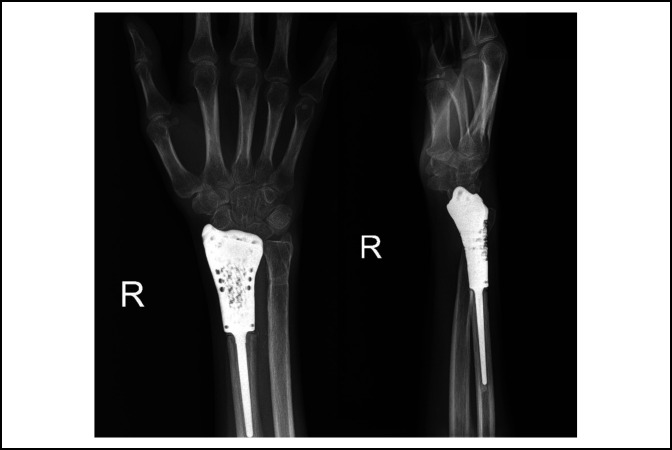
Postoperative x-ray of patient after 18-month follow-up in PA and lateral views.

The patient was evaluated postoperatively at 2, 6, and 12 weeks; 6 months; and on an annual basis with a physical examination and plain radiography. The resected radius length was 5.5 cm, and the tumor pathology showed negative margin. At the 18-month follow-up, the functional results revealed that the active range of motion of the operated wrist was 20° wrist extension, 10° wrist flexion, 10° supination, and 60° pronation; mean grip strength was 30 pounds (60% compared with the normal side). The patient was able to use her wrist for working and doing daily living activities with minimal pain (visual analog scale pain score = 2/10). Follow-up details of functional photographs of the patient are presented in Figure 7. The postoperative radiographs in Figure 8 shows the distal part of the implant, imitating the articular surface of the wrist joint. At 2 years of follow-up, the patient was contacted by phone only and not available for in-person assessment because of the COVID-19 pandemic. She reported a satisfied outcome because she was able to work properly without pain (visual analog scale pain score = 0/10) as shown in Video clip 1, http://links.lww.com/JG9/A115.

No early postoperative complications such as infection at the operative site, soft-tissue defect, immune rejection, periprosthetic fracture, nonunion, implant loosening, prosthetic dislocation, or tendon rupture were noted. At the final follow-up, no tumor recurrence and no signs of radiographic loosening were noted.

## Discussion

GCT is known to be an extremely locally aggressive bone tumor.^1,2,21^ Unlike Campanacci I and Campanacci II without pathological fracture, treatment for extended curettage in Campanacci III GCT of the distal radius is unpredictable because of the high recurrence rate of up to 70% from the residual tumor.^22^ However, the reconstruction of the defect that remains after an En bloc resection of the distal radius continues to pose a considerable challenge. The ideal goals of surgical reconstruction include a painless, stable, and mobile wrist with adequate grip strength. Reconstruction options should avoid postoperative complications and ensure low donor side morbidity. Several surgical options have been reported, yet no consensus exists about the best surgical reconstruction technique because of the limited literature and number of patients at present.

The first report of prosthesis replacement of distal radius after resection of recurrent GCT was by Gold in 1965.^23^ The prosthesis was made from Vitallium, which was associated with chronic inflammation. No osseous integration was noted for several endosteal and subperiosteal designs,^24^ and longevity was limited to 8 years. With the advancement of technology, several materials and designs produced different types of endoprostheses such as a bipolar hinge component and a stainless steel stem.^19^ The latest prostheses comprised acrylic and a long stem of stainless steel^23^ or alumina ceramic.^18^ Essentially, the benefits of endoprosthesis outweight other reconstruction techniques because it imitates the native distal radius and avoids delayed union and donor site morbidity. It also has the ability to repair large defects. Moreover, the emergence of 3D printing break throughs and constant innovation helps create a more individualized design to improve the functional outcome for patients. Nonetheless, long-term radioulnar subluxation^18^ and asymptomatic loosening^19^ have been reported in previous studies.

We feel that the restricted motion at the last patient visit is explained by the stiffness from the ligament reconstruction procedure. Midterm outcome from former clinical studies seem to provide a better range of motion. In 2012, Duan et al.^17^ reported a mean flexion/extension of 33.3°/46.7° and prono/supination of 72.3°/61.3° after allograft reconstruction in 15 patients. The current study of autograft with partial arthrodesis showed flexion/extension of 20°/20° to 30° and prono/supination of 30° to 80°/15° to 20°, which was wide in range and questionable according to the arthrodesis technique.^13^ Our goal for the radiocarpal and radioulnar ligament reconstruction is to prevent long-term complication of the joint subluxation that will affect grip strength and the functional outcome later on. During the surgery, we tightened the grafts slightly more than usual because delayed ligament laxity was expected. Therefore, the 2-year outcome does not provide an excellent range of motion. However, our reconstruction method provides painless and stable wrist that satisfies patient's activities which allows some motion and flexibility of her wrist. The limitation of this study is the 2-year follow-up only. Long-term functional outcome should be further evaluated.

Our 3D-printed custom-made endoprosthetic with multiligament reconstruction is an innovative and reasonable treatment method after en-bloc resection in GCT of the distal radius. It shows neither palmar subluxation nor distal radioulnar separation after surgery up until the 18-month follow-up. We feel that our results may be associated with the additional reconstruction step of the dorsal radiocarpal ligament, distal radioulnar ligament, and extensor retinaculum into our surgical technique to preserve all anatomical structures while avoiding donor-site morbidity compared with other methods.

## References

[1] Averill RM Smith RJ Campbell CJ: Giant-cell tumors of the bones of the hand. J Hand Surg Am 1980;5:39-50. 7365216 10.1016/s0363-5023(80)80042-6

[2] Athanasian EA Wold LE Amadio PC: Giant cell tumors of the bones of the hand. J Hand Surg Am 1997;22:91-98. 9018620 10.1016/S0363-5023(05)80187-X

[3] Campanacci ME: Bone and Soft Tissue Tumors, ed 2 New York, NY, Springer Verlag, 1999.

[4] Blackley HR Wunder JS Davis AM White LM Kandel R Bell RS: Treatment of giant-cell tumors of long bones with curettage and bone-grafting. J Bone Joint Surg Am 1999;81:811-820. 10391546 10.2106/00004623-199906000-00008

[5] Prosser GH Baloch KG Tillman RM Carter SR Grimer RJ: Does curettage without adjuvant therapy provide low recurrence rates in giant-cell tumors of bone? Clin Orthop Relat Res 2005:211-218. 15930941 10.1097/01.blo.0000160024.06739.ff

[6] Seradge H: Distal ulnar translocation in the treatment of giant-cell tumors of the distal end of the radius. J Bone Joint Surg Am 1982;64:67-73. 7054206

[7] Murray JA Schlafly B: Giant-cell tumors in the distal end of the radius. Treatment by resection and fibular autograft interpositional arthrodesis. J Bone Joint Surg Am 1986;68:687-694. 3722225

[8] Bickert B Heitmann C Germann G: Fibulo-scapho-lunate arthrodesis as a motion-preserving procedure after tumour resection of the distal radius. J Hand Surg Br 2002;27:573-576. 12475519 10.1054/jhsb.2002.0829

[9] Minami A Kato H Iwasaki N: Vascularized fibular graft after excision of giant-cell tumor of the distal radius: Wrist arthroplasty versus partial wrist arthrodesis. Plast Reconstr Surg 2002;110:112-117. 12087240 10.1097/00006534-200207000-00020

[10] Lackman RD McDonald DJ Beckenbaugh RD Sim FH: Fibular reconstruction for giant cell tumor of the distal radius. Clin Orthop Relat Res 1987:232-238. 3568485

[11] Weiland AJ Kleinert HE Kutz JE Daniel RK: Free vascularized bone grafts in surgery of the upper extremity. J Hand Surg Am 1979;4:129-144. 370187 10.1016/s0363-5023(79)80129-x

[12] Innocenti M Delcroix L Manfrini M Ceruso M Capanna R: Vascularized proximal fibular epiphyseal transfer for distal radial reconstruction. J Bone Joint Surg Am 2004;86:1504-1511. 15252100 10.2106/00004623-200407000-00021

[13] Legname M Barbary S Dautel G: Distal radius reconstruction using a split vascularized fibula. Two cases following giant cell tumor resection. Orthop Traumatol Surg Res 2011;97:762-765. 22000596 10.1016/j.otsr.2011.06.010

[14] Kocher MS Gebhardt MC Mankin HJ: Reconstruction of the distal aspect of the radius with use of an osteoarticular allograft after excision of a skeletal tumor. J Bone Joint Surg Am 1998;80:407-419. 9531209 10.2106/00004623-199803000-00014

[15] Bianchi G Donati D Staals EL Mercuri M: Osteoarticular allograft reconstruction of the distal radius after bone tumour resection. J Hand Surg Br 2005;30:369-373. 15951074 10.1016/j.jhsb.2005.04.006

[16] Szabo RM Anderson KA Chen JL: Functional outcome of en bloc excision and osteoarticular allograft replacement with the Sauve-Kapandji procedure for Campanacci grade 3 giant-cell tumor of the distal radius. J Hand Surg Am 2006;31:1340-1348. 17027797 10.1016/j.jhsa.2006.06.004

[17] Duan H Zhang B Yang HS: Functional outcome of en bloc resection and osteoarticular allograft reconstruction with locking compression plate for giant cell tumor of the distal radius. J Orthop Sci 2013;18:599-604. 23661178 10.1007/s00776-013-0394-1

[18] Hatano H Morita T Kobayashi H Otsuka H: A ceramic prosthesis for the treatment of tumours of the distal radius. J Bone Joint Surg Br 2006;88:1656-1658. 17159183 10.1302/0301-620X.88B12.17989

[19] Natarajan MV Chandra Bose J Viswanath J Balasubramanian N Sameer M: Custom prosthetic replacement for distal radial tumours. Int Orthop 2009;33:1081-1084. 19242692 10.1007/s00264-009-0732-2 PMC2899006

[20] Zhang S Xu MT Wang XQ Wang JJ: Functional outcome of en bloc excision and custom prosthetic replacement for giant cell tumor of the distal radius. J Orthop Sci 2015;20:1090-1097. 26329932 10.1007/s00776-015-0763-z

[21] Maloney WJ Vaughan LM Jones HH Ross J Nagel DA: Benign metastasizing giant-cell tumor of bone. Report of three cases and review of the literature. Clin Orthop Relat Res 1989:208-215. 2656024

[22] Oda Y Miura H Tsuneyoshi M Iwamoto Y: Giant cell tumor of bone: Oncological and functional results of long-term follow-up. Jpn J Clin Oncol 1998;28:323-328. 9703860 10.1093/jjco/28.5.323

[23] Gold AM: Use of a prosthesis for the distal portion of the radius following resection of a recurrent giant-cell tumor. J Bone Joint Surg Am 1957;39-A:1374-1380. 13481051

[24] Anusavice KJ Shen C Rawls HR: Phillips' Science of Dental Materials [E-Book]: New York, NY, Elsevier Health Sciences, 2014.

